# Genetic differences and longevity‐related phenotypes influence lifespan and lifespan variation in a sex‐specific manner in mice

**DOI:** 10.1111/acel.13263

**Published:** 2020-10-26

**Authors:** Rong Yuan, C. J. M. Musters, Yun Zhu, Tracy R. Evans, Yujie Sun, Elissa J. Chesler, Luanne L. Peters, David E. Harrison, Andrzej Bartke

**Affiliations:** ^1^ Department of Internal Medicine Geriatrics Research Southern Illinois University School of Medicine Springfield IL USA; ^2^ Department of Molecular Biology, Microbiology and Biochemistry Southern Illinois University School of Medicine Springfield IL USA; ^3^ Institute of Environmental Sciences Leiden University Leiden The Netherlands; ^4^ Department of Neurology Southern Illinois University School of Medicine Springfield IL USA; ^5^ The Jackson Laboratory Nathan Shock Centre of Excellence in the Basic Biology of Aging Bar Harbor ME USA; ^6^ Institute of Dermatology Chinese Academy of Medical Sciences and Peking Union Medical College Nanjing China

**Keywords:** antagonistic gene, female sexual maturation, IGF1, lifespan variation, maximum lifespan, sex difference in lifespan

## Abstract

Epidemiological studies of human longevity found two interesting features, robust advantage of female lifespan and consistent reduction of lifespan variation. To help understand the genetic aspects of these phenomena, the current study examined sex differences and variation of longevity using previously published mouse data sets including data on lifespan, age of puberty, and circulating insulin‐like growth factor 1 (IGF1) levels in 31 inbred strains, data from colonies of nuclear‐receptor‐interacting protein 1 (*Nrip1*) knockout mice, and a congenic strain, B6.C3H‐Igf1. Looking at the overall data for all inbred strains, the results show no significant difference in lifespan and lifespan variation between sexes; however, considerable differences were found among and within strains. Across strains, lifespan variations of female and male mice are significantly correlated. Strikingly, between sexes, IGF1 levels correlate with the lifespan variation and maximum lifespan in different directions. Female mice with low IGF1 levels have higher variation and extended maximum lifespan. The opposite is detected in males. Compared to domesticated inbred strains, wild‐derived inbred strains have elevated lifespan variation due to increased early deaths in both sexes and extended maximum lifespan in female mice. Intriguingly, the sex differences in survival curves of inbred strains negatively associated with age of female puberty, which is significantly accelerated in domesticated inbred strains compared to wild‐derived strains. In conclusion, this study suggests that genetic factors are involved in the regulation of sexual disparities in lifespan and lifespan variation, and dissecting the mouse genome may provide novel insight into the underlying genetic mechanisms.

## Graphical abstract

Among inbred strains, the lifespan variations of female and male mice are significantly correlated, suggesting the genetic co‐regulation of lifespan variation between sexes. IGF1 levels associate with lifespan variation and maximum lifespan in a sex‐specific manner. Importantly, female mice with lower IGF1 have longer maximum lifespan, while the male mice with higher IGF1 have longer maximum lifespan.

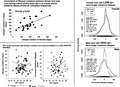

## 1 INTRODUCTION

Epidemiological studies of human lifespan data revealed that the sex difference in human longevity is one of the most robust features of human populations (reviewed in Austad & Fischer, 2016). According to information from 38 countries in the Human Mortality Database (http://www.mortality.org/), for all countries each year in the database, female life expectancy at birth exceeds that of males. Another evidence of greater female longevity is provided by data from the Gerontology Research Group (http://www.grg.org/Adams/Tables.htm), which shows that women comprise 90% of supercentenarians (individuals living to 110 years or longer). However, little about the underlying biological mechanisms is known, not only because of the complexity, but also due to the lack of a suitable animal model for studying this phenomenon. In fact, unlike in the human, common used laboratory models, worms, fruit flies, rats and mice, show there is no robust difference in longevity between sexes (Austad & Fischer, 2016).

Another important feature of human lifespan data identified by epidemiological studies is the inverse association between increased lifespan expectancy and decreased lifespan variation, which refers to the variation of age at death (Austad & Fischer, 2016; Raalte et al., 2018). Although lifespan variation is routinely reported along with other lifespan parameters, such as mean, median, and maximum lifespan, it is rarely analyzed. From a social‐economic perspective, understanding lifespan variation has profound impacts on the ability to predict an individual life expectancy, the organization and management of health care, and other policies (Raalte et al., 2018). For gerontologists, understanding variation of biomarkers of aging will improve the assessment of physiological dysregulation, which has attracted recent attention (Bartke et al., 2019). In aging research, the variation of lifespan is related to the resilience and robustness of resisting the exponentially increased death rate with increasing age; therefore, it may be used as a parameter to evaluate health span of a population and provide clues for delaying aging. Analysis of lifespan variation in mice subjected to anti‐aging interventions (pharmacological, dietary, or genetic modification) shows that the inverse association between lifespan and variation of individual age at death described in human cohorts is not consistently observed in these animals (Bartke et al., 2019). In fact, the impact of anti‐aging interventions on the lifespan variation of mice, defined as the median of absolute difference (MAD) between individual lifespan and the median lifespan of a population, is treatment‐dependent and sexually dimorphic (Bartke et al., 2019). These results indicate that biological mechanisms might be involved in regulating variability of lifespan.

In this paper, we used an extensive set of available data from several of our previous studies that investigated longevity‐related phenotypes in a variety of mouse strains, including inbred and genetically modified strains (Wang et al., 2018; Yuan et al., 2012, 2009). Our lifespan study of inbred strains was specifically designed to cover most of the genetic diversity of the mouse (*Mus musculus* species, including representatives of four subspecies). These data provided us with a unique opportunity to investigate the genetic regulation of lifespan variation—including conditions that generate sex differences in lifespan and lifespan variation—across a wide variety of well‐studied genotypes.

## 2 RESULTS

### 2.1 Similarities in longevity parameters between female and male mice

We previously reported significant differences in lifespan among inbred mouse strains (Yuan et al., 2012, 2009). Strain names and abbreviations are listed in the Supplementary Information, Table S1. In the current study, we first examined potential sex effects on lifespan (Table S2 and Figure S1). Although it is generally believed that female and male mice have different lifespans, our study comparing lifespan parameters of 1,851 female and 950 male mice, in aggregate from 31 mouse inbred strains, shows that any sex effect on survivorship (at 25%, 50%, and 75%) is small, including median lifespan, ranging from 0.8% to 1.7% (median test: z = −1.6844, *p* = 0.092; Table S2). The survival curves of female and male mice virtually overlap (log rank test: χ^2 ^= 0.3, *p* = 0.590; Figure S1).

The similarity in survivorship between sexes is also evident for the variation of longevity. In this study, to accommodate non‐normal data distributions, we used the MAD between individual and median lifespan to represent variability (Bartke et al., 2019). There was no sex effect on lifespan variation (variation test: z = −1.29, *p* = 0.200; Table S2).

### 2.2 Identification of individual strains having significant sex effects on lifespan

**FIGURE 1:**
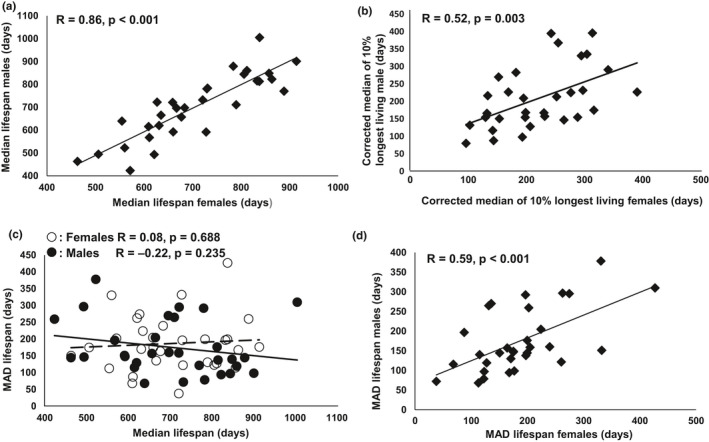
Association of median lifespan and MAD between sexes of inbred mouse strains. Median lifespan (a) and median of the longest 10% lifespan (b) are significantly correlated between sexes among inbred strains. There is no significant association between MAD and median lifespan among inbred strains (c). Female and male MADs are significantly associated (d)

Although the global analysis of our complete set of mice did not show a sex effect on longevity, genetic effects on possible sex differences may be found in individual strains. Therefore, we compared median and maximum lifespans (upper 10% of corrected lifespan), as well as female and male survival curves (Table S1). “Corrected lifespans” were calculated by subtracting the median lifespan of the group from the lifespan of individuals. It is well known that genetic factors play important roles in regulating lifespan. Hence, it is not surprising that median lifespan, as well as the median of the 10% longest corrected lifespans, significantly correlated between sexes among the inbred strains (Figure 1a,b).

The significance of the difference in maximum lifespan is determined by the combination of quantile regression and Fisher exact probability test, which takes the difference of sample size into account to avoid the potential bias caused by the inequality in sample size between groups. In this study, we defined a difference in longevity between sexes as a significant difference between survival curves (log rank test, *p* < 0.05), plus a significant difference in either median and/or maximum lifespan (*p* < 0.05). Males from strains 129S1, NOD.B10, and NZW had significantly greater longevity than females. Females from strains B10 and P mice had significantly greater longevity than males. Interestingly, female MRL mice had a significantly shorter median lifespan; however, females significantly outnumbered males in the longest 10% survivorship, indicating a higher death risk after the age of the median lifespan in males than in females of MRL.

### 2.3 Identification of strains with significant differences in lifespan variation

To identify inbred strains with significantly different MADs, we conducted a permutation test, defining the significantly higher or lower MAD as outside of the 2.5%–97.5% range of 1,000 permutations. For that, we pooled the mice per gender and drew per strain 1,000 times a random sample of the same size as the number of mice of that gender in that strain. Of each sample the MAD was calculated. This gave the expected distribution of MAD of that gender in that strain, which was used to estimate the 2.5%–97.5% range of MAD of that strain. It is worth noting that the permutation test was conducted according to the real sample size of each group; therefore, the difference in the sample size does not cause statistic bias. The significance level of the MAD differences between sexes of each strain was also determined by the same method. For the sex difference comparison, seven strains showed significantly different MADs between female and male (Table S3). In females, ten strains had significantly higher MADs than other strains; three strains had significantly lower MADs. In males, eight strains had significantly higher MADs than other strains; three strains had significantly lower MADs. In both sexes, strains C57L and MRL had significantly reduced MADs, while BUB, SWR, CAST, and WSB had significantly elevated MADs. WSB had the highest MAD in both females and males, and its MAD was also significantly different between sexes (Table S3).

In human populations, increased lifespan expectancy is consistently accompanied with decreased variation of age at death (Austad & Fischer, 2016; Raalte et al., 2018). In the current study, an examination of correlation showed that increased lifespan is correlated with reduced variation in males, while in females, MAD is slightly increased with longer median lifespan. However, none of the correlations is significant (*p* = 0.688 and 0.235 in females and males, respectively; Figure 1c). Interestingly, as shown in Figure 1d, the variation of longevity in female and male mice was significantly correlated (*R* = 0.59, *p* < 0.001).

### 2.4 Association of circulating IGF1 concentration, female reproductive maturation, and strain derivation with lifespan parameters

Our large strain study included analysis of a number of factors relevant to aging (Yuan et al., 2012, 2009). In the current study, we evaluated the potential association of three of these factors on lifespan variation: non‐fasting circulating IGF1 concentration, female reproductive maturation, and strain derivation.

#### 2.4.1 Association of IGF1 levels with lifespan parameters

**FIGURE 2:**
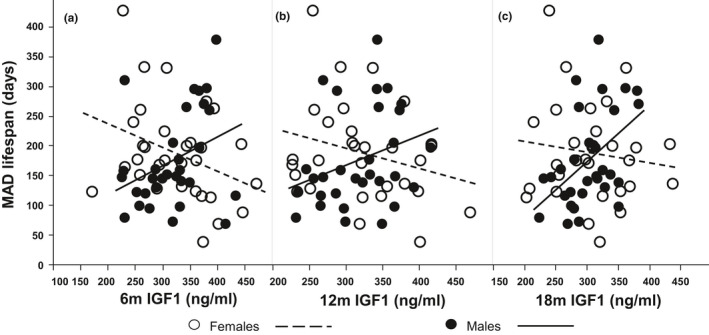
Association of MAD lifespan with median level of circulating IGF1 at ages of 6 (a), 12 (b), and 18 (c) months. At all three time points, the correlations are positive in females but negative in males. The correlations at 6 months in females and at 18 months in males are significant. The differences between sexes are significant at all three time points. The slopes and *p*‐values are listed in Table 1

Across inbred strains, we found that median level of IGF1 associated with the MAD lifespan, but in an age‐ and sex‐specific way. As shown in Figure 2a–c, in females, at all three time points, 6, 12 and 18 months, lower IGF1 associated with increased MAD and the association is significant at age of six months (*p* = 0.048, 0.144 and 0.464, respectively). In males, higher IGF1 at all three time points associated with reduced MAD and the association is significant at age of 18 months (*p* = 0.056, 0.106 and 0.010, respectively). Importantly, at all three ages, the difference between sexes is significant (*p* < 0.05, Table 1). These results indicate there might be sex‐specific effects of IGF1 on longevity.

**TABLE 1:** Association of MAD lifespan with circulating IGF1 levels at 6, 12, and 18 months.

**Age (month)**	**Female**		**Male**		**Diff. sexes**		**Model**	
	**Slope**	***p***	**Slope**	***p***	**Slope**	***p***	**adj. *R*^2^**	***p***
**6**	−0.40	**0.048**	0.49	0.056	0.89	**0.007**	0.08	0.051
**12**	−0.34	0.144	0.50	0.106	0.84	**0.032**	0.04	0.169
**18**	−0.18	0.464	0.95	**0.010**	1.13	**0.012**	0.08	0.058

*p*‐values in bold are smaller than 0.05.

**FIGURE 3:**
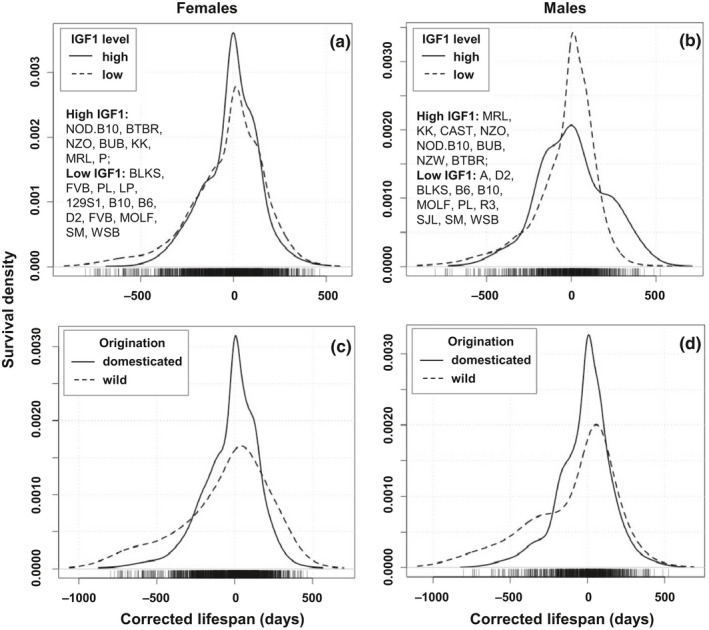
Comparison of survival density plots of strains with high and low IGF1 levels (a & b), and of the domesticated and wild‐derived inbred strains (c & d), females and male are shown separately. Strains with high and low level of IGF1 are listed in (a) and (b). Statistical comparisons of the groups are in Table 2. The *X*‐axes represent the corrected lifespan. The *Y*‐axes represent the probability that mice will survive up to the corrected lifespan. The probability density function is the kernel density estimation of R using default settings

We previously reported that IGF1 levels negatively associated with lifespan, and identified strains with significantly higher and lower IGF1 at age of six months in both females and males (Yang et al., 2009). To further investigate the relationship between IGF1 and lifespan, as well as the variation of lifespan, we compared the survival density plots (Figure 3a,b) and survival curves (Figure S2a,b) of the mice in the strains with significantly lower and higher IGF1. The lists of strains with respectively low and high IGF1 levels per sex are given in the figures. Log rank tests of the survival curves comparison show that these two groups of mice have significantly different longevities in both sexes (*p* < 0.001).

In females, the group with low IGF1 had an elevated death rate at young age. As shown in Table 2, the death risk estimation based on lognormal fit at the age of 180 days, before which mice are considered as young, is significantly higher in the mice with low IGF1 than that of the mice with high IGF1 (0.38% vs. 0.01%, *p* < 0.05). The median lifespan of female mice with low IGF1 level is significantly longer than that of the females with high IGF1 level (763 vs. 615.5 days respectively, *p* < 0.0001, Figure S2a, Figure 3a, Table 2). Moreover, comparing the density plots, when lifespan is corrected by median lifespan, low IGF1 females having longer maximum lifespans than high IGF1 females (Figure 3a). Among the mice of 10% longest corrected lifespan, 97 are low IGF1 mice and 31 are high IGF1 mice. Quantile regression test found the difference to be significant (*p* < 0.001, Table 2). The elevated rates of early deaths, as well as the extended median and maximum lifespan, result in the increased lifespan variation in the low IGF1 group compared to the high IGF1 group (z = −8.86, *p* < 0.001, Table 2).

**TABLE 2:** Comparison between sexes of IGF1 low versus high mice, and domesticated versus wild‐derived mice

Sex	Origination	*n*	Death risk at 180 days	Median	z	*p*	Max lifespan (10%)		Log rank		Variation			
							n	*p*	χ^2^	*p*	MAD	z	*p*	
**Female**	IGF1 Low	640	0.38% (0.23–0.62%)	763.0	−10.495	**<0.001**	**97**	**<0.001**	189.7	**<0.001**	245.37	−8.857	**<0.001**	
	IGF1 High	420	0.01% (0.00–0.03%)	615.5			31				134.18			
**Male**	IGF1 Low	345	0.62% (0.34–1.14%)	730.0	−5.030	**<0.001**	18	**<0.001**	17.5	**<0.001**	247.59	−1.046	0.296	
	IGF1 High	236	0.83% (0.42–1.64%)	615.0			**52**				218.68			
**Female**	Domest.	1,663	0.05% (0.03–0.07%)	694.0	−1.716	0.086	150	**<0.001**	24.96	**<0.001**	209.05	−7.397	**<0.001**	
	Wild	188	3.30% (1.90–5.50%)	737.5			**35**				278.73			
**Male**	Domest.	839	0.15% (0.10–0.26%)	708.0	−0.101	0.920	78	0.173	8.59	**0.003**	220.91	−5.374	**<0.001**	
	Wild	111	3.00% (1.50–6.00%)	715.0			15				314.31			

The IGF1 low and high strains are listed in Figure 3. Domest: domesticated inbred mice; Wild: wild‐derived inbred mice. *The death risk at age of 180 days is calculated based on lognormal fit using JMP 10. Numbers given in the parentheses are 95% confidence interval of the death risk. p‐values in bold are smaller than 0.05; n of max lifespan in bold indicates the group with significant higher number of longest living individuals.

In males, different from the females, there is no significant difference in MAD between the high and low IGF1 groups of male mice (z = −1.05, *p* = 0.296, Table 2). The death risk at 180 days is similar in the high and low IGF1 group (0.83% vs. 0.62%, respectively). The median lifespan is significantly extended in the males with low IGF1 than the males with high IGF1 (730 and 615 days respectively, *p* < 0.001). Surprisingly, among the 70 mice of 10% longest corrected lifespan, 52 mice belonging to seven strains are from the high IGF1 group. Only 18 mice are from the low IGF1 group. Quantile regression test shows the difference is significant (*p* < 0.001). These results indicate that male mice with high IGF1 have longer maximum lifespan.

It is interesting that our results show the opposite directions of the association between IGF1 with maximum lifespan between sexes (Figure S3), and the sexual differences are significant at all three time points (6, 12, and 18 months, Table S4). Importantly, in males, the IGF1 levels at 18 months have the most significant correlation with maximum lifespan compared with the other two time points (Table S4). In female mice, among the three time points, the IGF1 levels at six months correlate with maximum lifespan better than the other two time points (none of them is significant). Based on these results, it is reasonable to hypothesize that the IGF1 levels of male mice at old age and female mice at young age are important for predicting the maximum lifespan. The strains with high IGF1 levels in males at old age and in females at young age would favor males when comparing maximum lifespan between sexes, while the opposite is true for strains with low IGF1 in females at young age and in males at old age. According to our previous analyses (Yang et al., 2009), three strains, NOD, BUB, and NZO, fit the first scenario, and two strains, SM and WSB, fit the second scenario. In the first scenario, compared with females, males of NOD and BUB have significantly longer maximum lifespan, and NZO has a suggestively longer maximum lifespan (Table S1). As expected, female mice of SM and WSB have significantly longer maximum lifespan than males. Taken together, our results suggest that IGF1 plays an important role in the sexual disparity of the regulatory mechanisms of maximum lifespan.

We also examined the difference in IGF1 levels between sexes. Strains with significant sex disparities in IGF1 levels at three time points were identified (Table S5a–c). We also calculated the variation of IGF1 and examined the relationship between the lifespan MAD and IGF1 MAD, but found no significant association. Comparing the MAD of IGF1 between sexes, only a few strains had significant difference at the three time points (Table S5a–c).

#### 2.4.2 Association of the variation of age of vaginal patency (AVP) with lifespan variation and IGF1 levels

**FIGURE 4:**
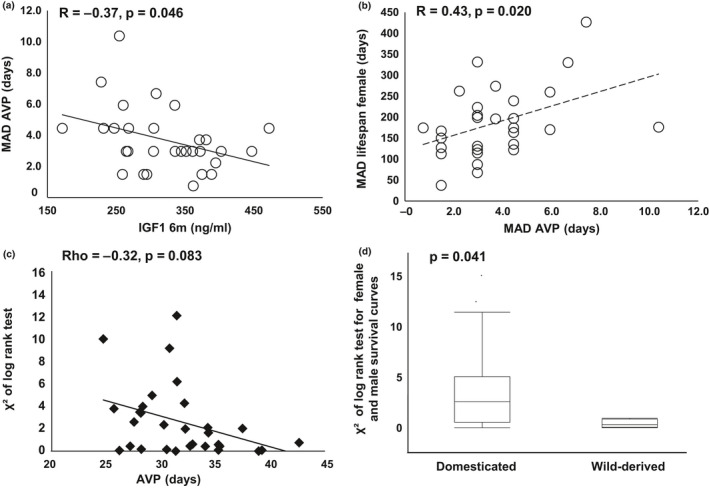
Association of sexual maturation with lifespan and IGF1 and the similarity of survival curves between sexes among inbred strains. Each marker represents one strain. Lower IGF1 at 6 months significantly correlates with Higher MAD of AVP (a). Higher MAD of AVP significantly correlates with higher MAD of lifespan of female mice (b). Earlier AVP suggestively correlated with higher χ^2^ (c). χ^2^, calculated by log rank test, represents the degree of difference between survival curves of female and male mice. Wild‐derived strains have significantly lower χ^2^ than that of domesticated inbred strains (d)

AVP, a biomarker of female sex maturation, like lifespan itself, is a life history trait. We have demonstrated that, in mice, delayed AVP associates with extended longevity in female mice (Yuan et al., 2012). In the current study, we evaluated variation of AVP and its relationship to IGF1 and variation of lifespan. We found that, as with the association between IGF1 and variation of lifespan, IGF1 levels at six months negatively associated with the MAD of AVP (*R* = 0.37, *p* = 0.046, Figure 4a). Furthermore, the variations of AVP and lifespan associated positively (*R* = 0.43, *p* = 0.020, Figure 4b), suggesting that IGF1 coordinately regulates life history traits throughout life.

#### 2.4.3 Differences in life history traits between domesticated and wild‐derived inbred strains

This study includes 27 domesticated inbred strains and four wild‐derived inbred strains CAST/EiJ, MOLF/EiJ, PWD/PhJ, and WSB/EiJ that represent four different subspecies *Mus musculus castaneus*, *Mus musculus molosinus*, *Mus musculus musculus*, and *Mus musculus domesticus*. More details of the originations of the strains involved in this study can be found in the website of The Jackson Laboratory (www.jax.org/strain). Although the median lifespan was not significantly different between the domesticated and wild‐derived inbred strains (Figure S4a,b, Table 2), the survival curves were significantly different for both females and males (*p* < 0.001 and *p* = 0.003 in females and males, respectively; Figure S2c,d, Table 2). Similarly, lifespan variations of domesticated and wild‐derived mice were also significantly different in both sexes (Table 2). Interestingly, for both sexes, wild‐derived inbred mice have a significantly greater death risk at age of 180 days, but only female wild‐derived mice exhibited a significantly greater maximum corrected lifespan (Figure 3c,d, Figure S2c,d, Table 2).

### 2.5 Association of AVP with lifespan disparity between female and male mice

Interestingly, we found a suggestive, negative correlation of AVP with the χ^2^ of the log rank test for sex differences in survival curves, suggesting that accelerated AVP increases the disparity of the survival curves between females and males (Rho = −0.32, *p* = 0.083, Figure 4C). This idea is supported by the comparison between domesticated and wild‐derived strains. The process of domestication and inbreeding favors the selection of alleles that accelerate sexual maturation, and we have previously reported that wild‐derived inbred strains have significantly delayed AVP compared to domesticated inbred strains (Yuan et al., 2012). In the current study, we found that wild‐derived inbred strains have significantly lower χ^2^ values for the comparison of survival curves of different sexes compared to the domesticated inbred strains (Figure 4D), indicating that the female and male survival curves are more similar in wild‐derived inbred strains than that in the domesticated inbred strains. These results suggest that strain differences in the sex effects on survival might have emerged during the process of domestication, accompanied with the accelerated female reproductive maturation.

**FIGURE 5:**
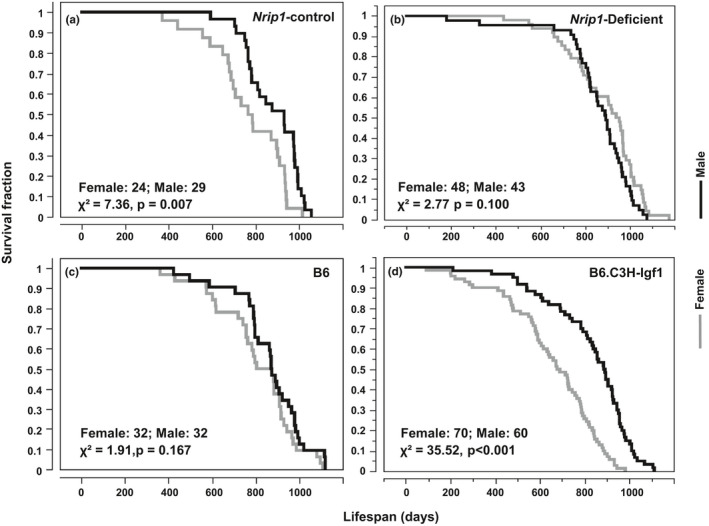
Altering age of sexual maturation alters the sex inequity in longevity. Significance of the difference in survival curves between sexes is determined by log rank test. In *Nrip1*‐control mice, the survival curves are significantly different between sexes (a). In the *Nrip1*‐deficient mice, no significant difference in survival curves between sexes is found and the χ^2^ is less than that of control mice, indicating the sex inequality is reduced in *Nrip1*‐deficient mice compared to the control mice (b). The survival curves of B6 mice are not significantly different between sexes (c). In the B6.C3H‐Igf1 colony, which carries an IGF1‐increasing allele derived from strain C3H on the B6 genetic background, there is significant difference in the survival curves of female and male mice (d). Mice are not significantly different between sexes (c). In the B6.C3H‐Igf1 colony, there is significant difference in the survival curves of female and male mice (d). Significance of the difference between survival curves is determined by log rank test

To verifying this hypothesis, in this study, we examined the relationship of AVP and lifespan disparity between sexes by using two genetically engineered mouse models (Yuan et al., 2012). In the nuclear‐receptor‐interacting protein 1 (*Nrip1*) deletion model, AVP is significantly delayed compared to *Nrip1*‐wild controls. In B6.C3H‐Igf1, a congenic strain carrying an IGF1‐increasing allele derived from C3H on B6 background, females had significantly higher IGF1 level and accelerated AVP than the parental B6 strain. In both studies, delayed AVP was associated with extended median and maximum lifespan of females, while males with the same genotypes did not show extended lifespan (Yuan et al., 2012). In the current study, we found that the alteration of age at sexual maturation alters the similarity of survival curves between sexes (Figure 5). In male and female control mice from the *Nrip1*‐control cohort, the lifespan curves are significantly different (χ^2 ^= 7.36, *p* = 0.007); however, deletion of *Nrip1*, including heterozygous and homozygous deletion, reduced the difference (χ^2^ = 2.77, *p* = 0.10). A comparison between female and male mice of the B6 strain, the parental strain of B6.C3H‐Igf1, showed no significant difference in the survival curves. Strikingly, in the B6.C3H‐Igf1 colony, the survival curves of female and male mice were dramatically different (χ^2^ = 35.52, *p* < 0.001).

## 3 DISCUSSION

Lifespan advantage in women has existed for as long as reliable demographic information is available, and is robust across diets, mating patterns, and environmental vagaries (reviewed in Austad & Fischer, 2016). Backtracking the historic demographic data, the sex bias in survival widened for mid‐ to late life survival in the very late 19^th^ and early 20^th^ centuries as a consequence of a rapid increase in cardiovascular disease in men that may be due to food superabundance and sex difference in smoking behavior (Beltran‐Sanchez et al., 2015). Interestingly, the widened female survival advantage can also be seen in 21th century by analyzing worldwide lifespan data (2007 world population data sheet, Population Reference Bureau of United States, www.prb.org). Comparing the shortest‐ and longest‐lived countries, the sex difference in the lifespan expectancy at birth (LEAB) is significantly lower in the short‐lived countries, in both terms of years or the percentage of the LEAB (data not shown), indicating the sex bias increased along with the extended lifespan, which mimics the changes that have been seen in western countries in the past two centuries (Beltran‐Sanchez et al., 2015; Beltran‐Sancheza et al., 2012). On the other hand, over the past 200 years, in the human population, increasing lifespan has been accompanied by a decline in the variation of lifespan, mostly resulting from strong reductions in infectious disease, maternal and child mortality, injuries, and, more recently, cancers (Raalte et al., 2018). Although the decreased longevity variation in a population can reduce uncertainty that may make it easier to plan a life course or social‐economic policy, it also presents a sign of a “ceiling effect,” as longevity approaches the value of maximal lifespan for a population with given genetic diversities and under specific environmental conditions. Although healthspan is one of the major focuses, aging researchers are eager to break through the “ceiling.” Using inbred mouse strains, our results may help reveal the underlying genetic mechanisms for these two long‐known, but not well‐understood, human longevity features. It should be noted that these inbred strains were selected to represent the major diversity in the genome of *Mus musculus* (Yang et al., 2009). It has been reported that genome diversity in *Mus musculus* is one to two orders of magnitude higher than the level of sequence diversity observed in the human population (Ideraabdullah et al., 2004). Therefore, investigating genetics of lifespan in mice may result in identifying novel mechanisms that could not be identified in human population.

### 3.1 Lifespan differences of female and male mice

In an analysis of 118 survival studies of laboratory mice, Austad *et al*. reported that mice display no robust or consistent sex difference in longevity (Austad, 2011). The current study supports these results when looking at our complete data set. This indicates, in general, that the longevity of male and female *Mus musculus* is similar. However, analysis of the individual mouse strains shows that a few strains do exhibit differences in longevity between sexes. Combining log rank tests, comparisons of median and maximum lifespan found that males have greater longevity in three strains (129S1, NOD.B10, and NZW), while female B10 and P mice have a significantly greater lifespan than males.

Understanding the sex inequality in lifespan is important for designing experiments, interpreting data of aging research, in which the mouse strains are involved. For instance, many embryonic stem cell lines used for generating transgenic and knockout mouse models are derived from strains within the 129S lineage (Linder, 2006), including 129S1, examined in the current report. Transgenic strains often backcross with B6, of which female and male mice have similar median and maximum lifespan, as well as the survival curves. These strain‐specific features in lifespan parameters may be a source of phenotypic difference among aging studies that use different genetic backgrounds, and a source of bias, when the genetic background is mixed. Importantly, the differences in the lifespan of female and male 129S1 mice indicate the potential different pathological mechanisms in determining the aging rate and longevity in different sexes of this strain. Therefore, when 129S1 genetic background is involved in an aging study, its sexual disparities need to be considered in experimental design and data interpretation.

Sex inequality in lifespan, in some cases, may reflect the sex‐related pathological difference. For instance, in nonobese diabetic (NOD) mice, alleles at multiple genetic loci, including the major histocompatibility complex (MHC), prompt the development of autoimmune diabetes. NOD.B10 is a congenic strain, in which the MHC *H2^b^* is substituted the nonpathogenic allele from the B10 strain on the NOD background. Although NOD.B10 does not develop insulitis, cyclophosphamide‐induced diabetes, or spontaneous diabetes, it does develop autoimmunity as shown by the extensive lymphocytic infiltration in pancreas and submandibular glands (Wicker et al., 1992). It has been shown that female mice are more susceptible to autoimmune disorder and androgen treatment could reduce the risk (Fox, 1992; Voskuhl, 2011). Whether or not the difference in the susceptibility to autoimmune disorders is involved in the regulation of sex disparity in lifespan needs to be further investigated. Importantly, although the differences in the lifespan parameters between sexes of one inbred strain can provide new clues for investigating specific pathologic factor(s) that may have sex‐specific impacts on health, from a broader genetic perspective, utilizing a well‐defined heterogeneous colony, such as four‐way cross mice (Miller et al., 1999) or diversity outbred mice (Churchill et al., 2012), is necessary to further explore the genetic regulation of sex‐dimorphic lifespan.

### 3.2 Lifespan differences of female and male mice may have evolved from domestication

The difference in lifespan between sexes may be related to the process of domestication. Most of the inbred strains were developed from mice that have been domesticated for thousands of years and raised as “fancy” mice for hundreds of years (Silver, 1995). In this study, four strains (CAST, PWD, WSB, and MOLF) were developed from wild‐caught mice that were housed in laboratory for only a few generations (Beck et al., 2000; Silver, 1995; Tucker et al., 1992). The χ^2^ of the survival curve comparison showed that wild‐derived inbred strains have significantly higher similarity between sexes than that of the domesticated inbred strains, suggesting that some of the differences between sexes might have evolved during the process of domestication.

Previous studies revealed that the processes of domesticating and inbreeding have profound effects on mice development and aging. Bronson F. H. reported that compared to wild‐caught mice, domesticated mice (not inbred) had significantly earlier onset of fertility, accompanied with greater food consumption, growth rate, and final body weight, but depressed locomotor activity (Bronson, 1984). Miller R. A. observed a similar result in the comparison between heterogeneous stock produced by crossing four inbred strains and wild stocks (wild‐caught mice) (Miller et al., 2002). These results indicate the domestication process does accelerate female sexual maturation. The acceleration of female sexual maturity by domestication is further verified in our previous report, in which AVP of domesticated inbred strains is significantly accelerated than that of wild‐derived inbred strains (Yuan et al., 2012). We also showed that the alleles responsible for delayed development might also be involved in extending lifespan (Yuan et al., 2012).

In the current study, we found that across the inbred strains, later sexual maturation measured by AVP, suggestively associated (*p* = 0.083) with reduced difference between sexes among the inbred strains, suggesting the delayed female sexual maturation is accompanied with increased similarity between female and male longevity. Therefore, these results raise an interesting hypothesis that the alleles delaying sexual maturation may not only extend longevity but may also reduce the sexual differences of lifespan. The observations in *Nrip1* knockout and B6.C3H‐Igf1 mouse colonies strongly support this hypothesis. Sexual maturation is delayed in *Nrip1*‐deficient females and accelerated in B6.C3H‐Igf1 females (Yuan et al., 2012). In the current study, we found that along with the delayed AVP, the sex difference in lifespan diminished in the NRIP1‐deficient mice, compared to littermate controls. B6.C3H‐Igf1 females consistently have earlier AVP than B6, and the survival curve of females is obviously separated from the curve of males. Meanwhile, among the control B6 mice, there is no significant difference in the survival curves between sexes. This hypothesis is also supported by previous studies. Miller R. A. and his colleagues reported that, compared to heterogeneous stock produced by crossing four inbred strains, two of three wild‐derived populations, with little domestication and no inbreeding, have delayed female sexual maturation. The difference between sexes in mean lifespan of the outbred mice is 18.5% (723 vs. 909 days, female and male respectively), while the difference is 1% or less for two of three wild‐derived populations (Miller et al., 2002), thus indicating the non‐domesticated, wild‐caught mice carry alleles that delay sexual maturation and reduce the difference in lifespan between sexes.

Interestingly, observations in SM mice further support that the selection of developmental traits can affect sexual maturation and lifespan difference between sexes. SM mice were selectively bred for small body weight during inbreeding (MacArthur, 1944). Genome haplotype analysis found that the SM strain shares the same haplotypes as the wild‐derived strains at two of three quantitative trait loci of AVP, which is not seen in other domesticated inbred strains. AVP of SM females is one of the latest and is significantly later than in 14 domesticated inbred strains (Yuan et al., 2012). Interestingly, in this study, we found that survival curves of SM females and males are more similar than 24 of 27 domesticated inbred strains (Table S1).

It is worth pointing out that AVP is one of the markers of female sexual maturation. Other commonly used markers include age of vaginal cornification and onset of cyclicity. It has been reported that among three inbred strains, B6, D2, and C3H, differences in the onset of vaginal opening and first vaginal cornification did not parallel those for the onset of cyclicity, indicating that the set of genes specifying the timing of vaginal opening and first vaginal cornification differs from those specifying the onset of cyclicity (Nelson et al., 1990). A systematic study of the biological markers of female sexual maturation, as well as the biomarkers of female reproductive life traits, such as age of first litter and first successful weaning, may provide more valuable data to investigate the relationship between female reproduction and aging.

### 3.3 Examining lifespan variation among inbred strains provides clues for identifying novel mechanisms of extending longevity

In the human population, increased lifespan is consistently accompanied by reduced variation (Raalte et al., 2018). In our study, we found no significant correlation between MAD and median lifespan. In male mice, there was a slight inverse correlation, while in female mice, MAD was slightly increased with longer median lifespan. This pattern was also found in the mouse colonies used for the NIH Interventions Testing Program (ITP; Bartke et al., 2019). Examining each of the strains, the current study showed that there is considerable difference in MAD among the inbred strains. In female mice, the most significant difference in MAD was found between WSB (a wild‐derived inbred stain), and C57L mice. WSB females have 16% longer median lifespan than that of C57L females. However, the MAD of WSB is 11.3 times higher than the MAD of C57L. Males from another wild‐derived inbred strain, CAST, had an 18.3% shorter median lifespan than MRL, but its MAD was 5.5 times higher than that of MRL. Importantly, the significant correlation of MAD between females and males indicates that, in addition to the median and maximum lifespan, genetic factors are also involved in the regulation of lifespan variance.

Interestingly, as shown in Table 2, the variation of longevity in wild‐derived strains is significantly higher than those of domesticated inbred strains. Comparison of survival curves showed that the increased lifespan variation of wild‐derived inbred mice resulted from the increased death at an early age of both sexes as well as extended maximum lifespan in females. Although further investigation is needed, combining the results of this study with the knowledge that deletion of *Nrip1* delays AVP and extends lifespan, and that the upregulation of IGF1 accelerates AVP but reduces lifespan (Yuan et al., 2012), suggests that antagonistic alleles selected by domestication, which favor early development but are detrimental to health at old age, are responsible for the alterations in the lifespan variation. Testing this hypothesis may provide innovative approaches for identifying novel aging genes along with clues to develop new interventional methods to delay aging and extend maximum lifespan. These studies will require use of wild‐derived (instead of the domesticated) animals because the responsible alleles may have been lost in domesticated inbred strains. Importantly, our results also suggest a need for caution of the potential risk of increasing early deaths by interventions of extending longevity. Supporting this concern, Harper J. M. and his colleagues reported that dietary restriction in wild‐derived heterogeneous male mice increased early death but extended maximum lifespan (Harper et al., 2006). Consistent with results in mice, dietary restriction in short‐lived fish *Nothobranchius furzeri* extended maximum lifespan in both wild‐derived and inbred lines, but increased the early mortality rate in the wild‐derived line (Terzibasi et al., 2009). A meta‐analysis across species found that early life dietary restriction increases variability in longevity (Senior et al., 2017). In addition, for evaluating early death, it may be crucial to include data from pups before weaning, and even prenatal embryos. In our previous report of extended lifespan in *Nrip1* knockout mice, we did not find significantly increased early deaths (Yuan et al., 2012). However, we recently found that the number of successfully weaned *Nrip1* homozygous knockout females, produced by the parents with *Nrip1* heterozygous knockout, is only 30% of the numbers estimated by the basic laws of genetics, indicating higher rates of early death before weaning and/or failure of embryo development (Yuan, unpublished data).

### 3.4 IGF1 is involved in regulating sexual differences of lifespan parameters

Our previous studies suggest that IGF1 is a co‐regulator that mediates the trade‐off relationship between the age of female sexual maturation and longevity. Strains with lower IGF1 have delayed AVP and greater longevity (Yuan et al., 2012). Delayed puberty and markedly extended longevity are characteristic of several genetically growth hormone (GH)‐deficient or GH‐resistant mice, in which circulating IGF1 levels are profoundly suppressed (reviewed in Aguiar‐Oliveira & Bartke, 2019; Bartke, 2019). The current study shows that lower IGF1 levels associate with increased variation of AVP, which positively associates with lifespan variation of female mice. Therefore, not surprisingly, lower IGF1 levels associated with extended maximum lifespan but increased variation in female mice.

However, this correlation is completely opposite in the male mice, which is one of the interesting findings in the current study. At all three time points, the correlations are in opposite directions and the differences between sexes are significant (*p* < 0.05), demonstrating the robustness of this phenomenon. Examining the survival curves and survival density plots of mice with high and low IGF1 levels helps us understand this pattern. In both sexes, mice with lower IGF1 have extended median lifespan than mice with higher IGF1. However, female mice with lower IGF1 have increased death rate at young age and longer maximum lifespan than the females with higher IGF1, mimicking the pattern seen in the comparison between wild‐derived inbred females and the domesticated‐derived inbred females. In the males, mice with lower IGF1 do not have increased death rate at young age, but have significantly shortened maximum lifespan than that of mice with higher IGF1. These results suggest that IGF1, a key component of nutrient‐sensing pathways, regulates aging and longevity in a sex‐specific manner. Evidence for sexual dimorphism in the impact of nutrient‐sensing pathways on longevity is provided also by studies of mice with deletion of genes related to insulin/IGF1 signaling (IIS) and mammalian target of rapamycin (mTOR) signaling. Heterozygous IGF1 receptor inactivation (IGF1R+/‐) and deletion of ribosomal S6 kinase 1 or insulin receptor substrate 1 genes extend longevity of female, but not male mice (Holzenberger et al., 2003; Selman et al., 2008, 2009). While the magnitude of the extension of longevity in IGF1R+/‐ mice was strongly dependent on the genetic background, the sex specificity of this effect was seen consistently in different studies (Bokov et al., 2011; Holzenberger et al., 2003; Xu et al., 2014). Differences in the magnitude of life extension in IGF1R +/‐ mice seen in different studies were related and presumably due to differences in the constitutive level of activation of the IGF pathways in the employed strains (Xu et al., 2014). Moreover, treatment of 18‐month‐old mice with a monoclonal antibody to IGF1 receptor extended median lifespan in females only and improved health span preferentially in females (Mao et al., 2018).

Interestingly, major sex differences are also seen in the life‐extending effects of pharmacologic interventions targeting glucose homeostasis or other metabolic parameters related to IIS (Garratt et al., 2017; Harrison et al., 2014, 2019). In the ITP studies, chemicals that extending longevity in male mice also reduced early death of the male mice, thereby reducing lifespan MAD (Bartke et al., 2019). In female mutant mice with reduced GH signaling, dietary restriction increased early death while increasing the maximum lifespan; therefore, the lifespan variation also increased (Bartke et al., 2019). These results suggest that although suppressing the IGF1 pathway might be a common mechanism for extending median lifespan of female and male mice, the underlying mechanisms might be different between sexes. More importantly, the approaches of extending maximum lifespan by manipulating IGF1 signal might be different between sexes.

Furthermore, the genetic diversities in IGF1 pathway regulations may all have impacts on the IGF1 signaling. It will be interesting to determine whether or not the IGF1 level positively correlates with the IGF1 signaling across the inbred strains. Fully addressing these questions is out of the scope of the current report. However, it is worth noting that the significant opposite correlation between IGF1 and maximum lifespan of different sexes indicates the importance of sexual disparity in the regulatory mechanisms instead of the genetic diversity in IGF1 pathway genes.

In addition, regulatory mechanisms of IGF1 signaling may reach beyond genetics. For instance, decreased total energy intake and protein‐calorie malnutrition may result in growth hormone (GH) resistance, in which the IGF1 level is reduced although the GH level is upregulated (Fazeli & Klibanski, 2014). Interestingly, recent studies have linked undernutrition, gut microbiota, and the signal of GH/IGF1 axis. In humans, undernutrition has been associated with major alterations in gut microbiota, which correlate with retarded juvenile growth (Subramanian et al., 2014). In mouse models, it has been reported that short‐chain fatty acids (SCFAs), produced when microbiota ferment fiber, also induce IGF1 (Yan et al., 2016). Because of its impact on human metabolism and immune function, the gut microbiome has been proposed as a possible determinant of healthy aging (Caracciolo et al., 2014; Claesson et al., 2012). It will be interesting to determine if the alteration of IGF1 signaling is involved in the mechanisms of extending healthspan *via* the improvement of gut microbiota.

## 4 CONCLUSION

This study reveals that variation of lifespan might be, at least partly, genetically regulated. The differences in lifespan traits between domesticated and wild‐derived inbred strains provide a unique model to investigate the genetic regulation of the variation and the sex disparity of lifespan. Importantly, the results show that accelerated sexual maturation in females, which evolved during the process of domestication, is accompanied by the increased sexual disparity of longevity. This may provide a novel angle to investigate the mechanisms of sexuality of lifespan in the human population. Importantly, the specific pattern of survival curves of the female wild‐derived inbred mice, in which the extended maximum lifespan is accompanied by an increased death rate at young age, emphasizes the trade‐off relationship between development and aging. This may lead to identification of anti‐aging alleles carried by wild‐derived inbred mice that will help break through the “ceiling” of maximum lifespan. Finally, the sex disparities, including the opposite correlations between IGF1 with the variation of lifespan, as well as the different effects of reduced IGF1 on the rate of early deaths and the maximum lifespan, indicate the mechanisms and approaches of extending longevity, especially the maximum lifespan, should be sex specific.

## 5 MATERIALS AND METHODS

### 5.1 Mice and data sets

All mice were housed in The Jackson Laboratory, and housing conditions have been previously described (Yuan et al., 2012, 2009). The longevity data in the current study contain three sets of data. The first data set of inbred strains has been reported (Yang et al., 2009). In that report, any mice that died due to unnatural causes, such as fighting or mishandling, were included in longevity study as censored data. However, since the major focus of this study is the variation of natural lifespan, we excluded the censored data here. Additionally, we also excluded the AKR/J mice that were used for the previous report, because the AKR/J mice have limited relevance to aging research, with most of them dying before 300 days of age due to leukemia or lymphoma (Herr & Gilbert, 1983). Early death due to specific malignant tumors likely increases the “background noise” in investigating aging‐related lifespan variation. The second set of data contains mostly female mice of the same inbred strains, along with a few male mice, to increase the statistical power on the base of the first set of data. The information of these mice is in the Table S6a. The first and second sets of data are labeled as “LG1” and “LG2.” In total, 1,851 female and 950 male mice are involved in the current report. The average numbers of mice of each strain are 59.7 ± 7.4 females and 30.6 ± 2.7 males. The strain names and their abbreviations, as well as the numbers of each strain, are listed in Table S1. The third set of data is the lifespan data of *Nrip1* knockout and B6.C3H‐Igf1 colonies that have also been previously reported (Yuan et al., 2012). Whole cohort information is provided in the supplemental material (Table S6b,c).

The current study also revisited the data that have been previously reported regarding non‐fasting IGF1 levels and AVP of the inbred strains, B6.C3H‐Igf1, and *Nrip1* knockout colonies (Yuan et al., 2012; Yuan et al., 2009). The details of measuring IGF1 concentration and determining AVP were fully described in the previous publications (Yuan et al., 2012, 2009). The IGF1 data are available in Mouse Phenome Database at The Jackson Laboratory (https://phenome.jax.org/projects/Yuan1; Bogue et al., 2016).

### 5.2 Statistical analysis

Survival curves were drawn using the Kaplan–Meier method. Differences between curves were analyzed by the log rank test. The death risk at age of 180 days is calculated based on lognormal fit. These analyses were conducted by using JMP 10 (SAS Inst. Cary, NC).

All other analyses were done using R software version 3.6.0 (R Core Team, 2019).

For studying lifespan and variation in lifespan, we calculated the median longevity of the females and males of all mice combined across strains, and of the females and males per strain. We chose median instead of mean because we could not assume that the variation in lifespan was normally distributed. For the variation in lifespan, we calculated the MAD, that is, the Median Absolute Deviation, a robust metric fit for non‐normal distributions (Leys et al., 2013). All median and MAD per strain were calculated with the function *describeBy()* of the package *psych* (Revelle, 2018). For testing the difference in median lifespan between groups, we used the function *median_test()*, and for testing the difference in variance of lifespan, we used the function *fligner_test()*; both functions are from the package *coin* (Strasser & Weber 1999). Density plots were drawn with the *densityPlot()* function of the package *car* (Fox & Weisberg, 2019).

For testing the difference in maximum lifespan, we applied the quantile regression approach, choosing the 10% longest living individuals, combined with the Fisher exact probability test as recommended by Wang et al. (2004). To determine the longest living mice, we followed the procedures developed by Redden *et al*. whereby one estimates the percentiles of a distribution using conventional quantile regression in a reduced predictive model that does not include the independent variable of interest (Redden et al., 2004). So, for example, one selects the 10% mice with the longest lifespan of the complete data set, then counts the number of males and females in this selection, and then tests whether the proportion of males and females is equal to the proportion in the complete data set. For that test, we used the *fisher*.*exact()* function of the *exact2X2* package (Fay, 2010). For assessing the longest living individuals of a group of mice, we first subtracted the median lifespan of the lifespans of the individual so that the test of the difference in maximum lifespan was not affected by the median of the group. We called this the “corrected lifespan.” For calculating the correlation between the maximum lifespan of females and males, we took the median of the 20% longest living females and males after correction for the median lifespan.

To assess whether the MADs of the females and males per strain were exceptional high or low as compared to the MAD of all the females and males, we performed a permutation test. For that, we took per strain 1000 times a random sample from all females or males of the size equal to the number of females or males of that strain. For each of these samples, we calculated the MAD. This gave us the distribution of 1000 random MADs per gender per strain. Then we assessed whether the observed MAD of the females and males per strain was below the 2.5% quantile or above the 97.5% quantile of these distributions. If so, we regarded the MAD as either extremely low or extremely high (gray, respectively bold in Table S3).

For calculating the slope, that is, the regression coefficient, per gender between median IGF1 level at different times of measurement and MAD of strains, we applied a linear model, including the interaction factor between median IGF1 level and sex, using the function *lm()*.

For the correlations between any two variables, we calculated the Pearson's R, unless the residuals were not distributed normally. In that case, we calculated Spearman's Rho with the function *cor*.*test()*.

## CONFLICT OF INTEREST

The authors declare that they have no conflicts of interest.

## AUTHOR CONTRIBUTIONS

RY designed and managed the study, contributed to data analysis, interpretation of results,and manuscript. CJM carried out the data analyses, contributed to results interpretation and manuscript. YZ, TE, and YS contributed to the manuscript. LLP, DEH, and AB contributed to experiment design and manuscript.

## Data Availability

Lifespan data of inbred strains, NRIP1 deficient mice as well as B6.C3H‐Igf1 and control mice are provided in Table S6a‐c. The circulating IGF1 data of inbred strains at three time points are accessible in Mouse Phenome DataBase at The Jackson Laboratory (https://phenome.jax.org/projects/Yuan1).
