# Supplementary figures and images for: Genetic differences and longevity‐related phenotypes influence lifespan and lifespan variation in a sex‐specific manner in mice
Source: Aging Cell. 2020 Oct 26;19(11):e13263. doi: 10.1111/acel.13263 (PMC7681063; doi:10.1111/acel.13263)

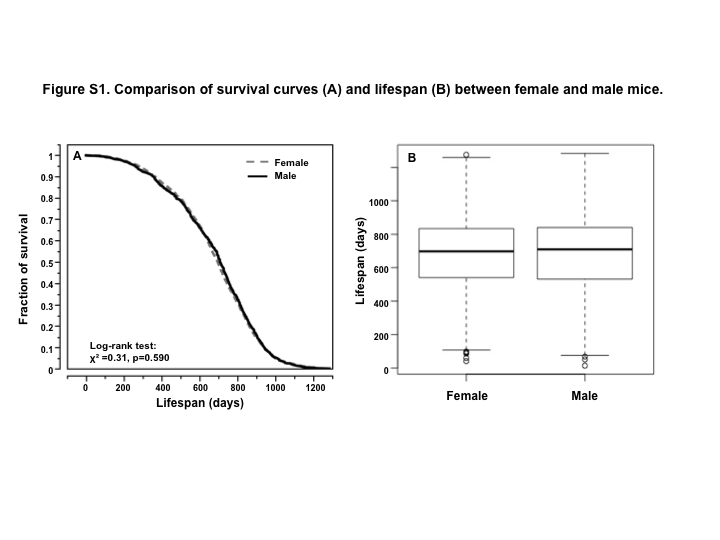

Supplement: Supplementary file 1 [file ACEL-19-e13263-s001.tiff]

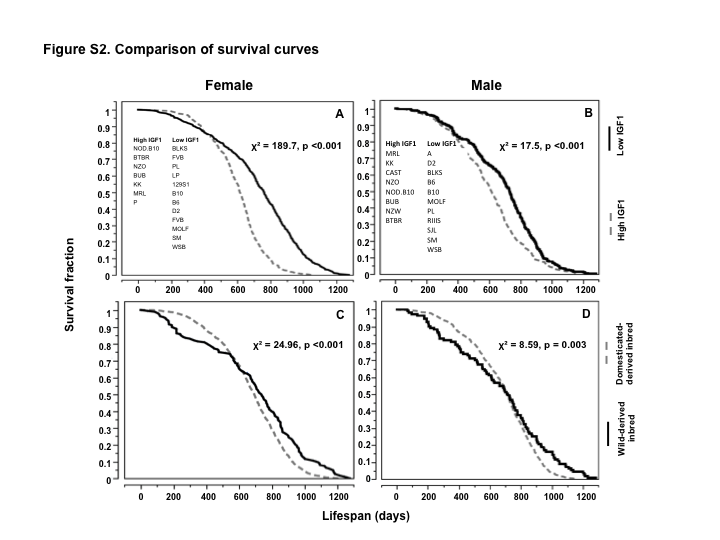

Supplement: Supplementary file 2 [file ACEL-19-e13263-s002.tiff]

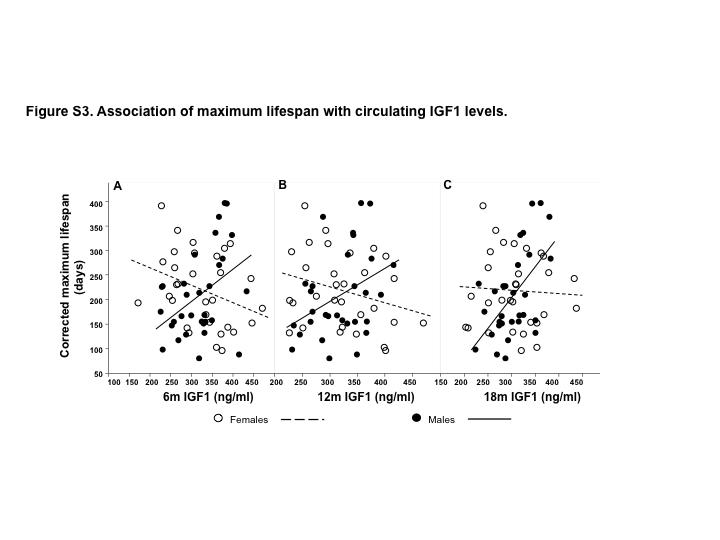

Supplement: Supplementary file 3 [file ACEL-19-e13263-s003.tiff]

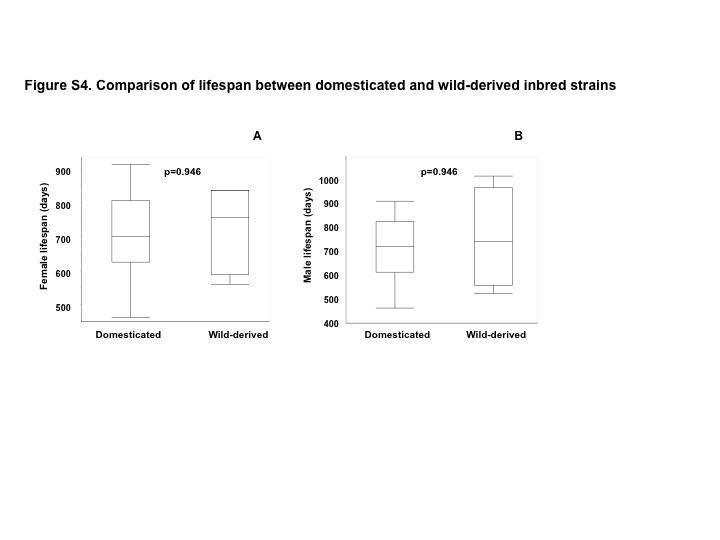

Supplement: Supplementary file 4 [file ACEL-19-e13263-s004.tiff]
