# Supplementary material for: Genetic differences and longevity‐related phenotypes influence lifespan and lifespan variation in a sex‐specific manner in mice
Source: Aging Cell. 2020 Oct 26;19(11):e13263. doi: 10.1111/acel.13263 (PMC7681063; doi:10.1111/acel.13263)
Supplement: Supplementary file 5 [file ACEL-19-e13263-s005.docx]

**Table S1. Lifespan comparison between sexes**

| **Strain** | **Abbreviation** | **Sample size** | | **Median lifespan** | | | | **Max lifespan (10%)** | | | **Sex difference in survival curves** | |
| --- | --- | --- | --- | --- | --- | --- | --- | --- | --- | --- | --- | --- |
|  |  | **Female** | **Male** | **Female** | **Male** | **z** | **p** | **n females** | **n males** | **p** | **χ²** | **p** |
| 129S1/SvImJ | 129S1 | 64 | 32 | 784 | 879 | -2.15 | **0.030** | 9 | 1 | 0.157 | 4.26 | **0.039** |
| A/J | A | 64 | 30 | 631 | 620 | 0.30 | 0.760 | 6 | 2 | 1.000 | 0.19 | 0.664 |
| BALB/cByJ | cBy | 64 | 32 | 790 | 710.5 | 1.44 | 0.150 | 3 | **7** | **0.014** | 2.49 | 0.115 |
| BTBR<+>tf/J | BTBR | 64 | 32 | 611 | 567.5 | 1.29 | 0.200 | 2 | **8** | **0.002** | 0.73 | 0.392 |
| BUB/BnJ | BUB | 55 | 25 | 621 | 493 | 0.57 | 0.570 | 2 | **6** | **0.010** | 0.07 | 0.796 |
| C3H/HeJ | C3H | 61 | 32 | 659 | 721 | -1.24 | 0.210 | 7 | 3 | 1.000 | 6.22 | **0.013** |
| C57BL/10J | B10 | 64 | 27 | 888.5 | 770 | 2.44 | **0.010** | 9 | 0 | 0.053 | 15.12 | **<0.001** |
| C57BL/6J | B6 | 61 | 32 | 914 | 901 | 0.36 | 0.720 | 6 | 4 | 0.732 | 0.54 | 0.464 |
| C57BLKS/J | BLKS | 56 | 32 | 863.5 | 822.5 | 0.44 | 0.660 | 5 | 4 | 0.718 | 0.01 | 0.919 |
| C57BR/CDJ | BRCD | 64 | 32 | 857.5 | 848.5 | 0.43 | 0.670 | 6 | 4 | 0.727 | 0.57 | 0.45 |
| C57L/J | C57L | 64 | 32 | 721 | 732 | -1.29 | 0.200 | 7 | 3 | 1.000 | 0.55 | 0.458 |
| CAST/EiJ | CAST | 35 | 21 | 560 | 522 | 0.17 | 0.860 | 3 | 3 | 0.661 | 0 | 0.997 |
| CBA/J | CBA | 62 | 32 | 635 | 665 | -0.58 | 0.560 | 8 | 2 | 0.486 | 0.22 | 0.64 |
| DBA/2J | D2 | 64 | 32 | 683.5 | 697.5 | -0.43 | 0.670 | 9 | 1 | 0.157 | 2.53 | 0.112 |
| FVB/NJ | FVB | 61 | 25 | 728 | 591 | 1.52 | 0.130 | 6 | 2 | 1.000 | 4.73 | **0.03** |
| KK/H1J | KK | 64 | 32 | 609.5 | 615 | -0.43 | 0.670 | 5 | 5 | 0.293 | 2.94 | 0.087 |
| LP/J | LP | 53 | 32 | 833 | 815 | 0.64 | 0.520 | 8 | 1 | 0.144 | 2.05 | 0.152 |
| MOLF/EiJ | MOLF | 36 | 32 | 677 | 657 | 0 | 1.000 | 5 | 2 | 0.434 | 0.56 | 0.455 |
| MRL/MpJ | MRL | 62 | 32 | 554.5 | 639 | -2.31 | **0.020** | **10** | 0 | **0.014** | 4.98 | **0.026** |
| NOD.B10Sn-H2<b>/J | NOD.B10 | 64 | 31 | 667 | 696 | -0.29 | 0.770 | 3 | **7** | **0.013** | 4.35 | **0.037** |
| NON/ShiLtJ | NON | 61 | 32 | 806 | 844.5 | -1.53 | 0.130 | 4 | 5 | 0.266 | 12.51 | **<0.001** |
| NZO/H1LtJ | NZO | 60 | 31 | 571.5 | 423 | 1.91 | 0.060 | 3 | 6 | 0.058 | 2.74 | 0.098 |
| NZW/LacJ | NZW | 64 | 32 | 729.5 | 780.5 | -1.29 | 0.200 | 3 | **7** | **0.014** | 7.75 | **0.005** |
| P/J | P | 51 | 29 | 660 | 592 | 2.54 | **0.010** | **8** | 0 | **0.046** | 11.48 | **0.001** |
| PL/J | PL | 64 | 32 | 463 | 462.5 | 0 | 1.000 | 8 | 2 | 0.488 | 0.79 | 0.374 |
| PWD/PhJ | PWD | 64 | 27 | 837.5 | 813 | 0.62 | 0.540 | 7 | 2 | 0.720 | 0.94 | 0.333 |
| RIIIS/J | R3 | 64 | 32 | 812 | 860 | -1.29 | 0.200 | 8 | 2 | 0.488 | 5.34 | **0.021** |
| SJL/J | SJL | 62 | 34 | 506 | 494 | 0 | 1.000 | 7 | 3 | 1.000 | 2.63 | 0.105 |
| SM/J | SM | 64 | 31 | 731 | 783 | -1.60 | 0.110 | **10** | 0 | **0.028** | 0.13 | 0.719 |
| SWR/J | SWR | 62 | 32 | 627 | 722 | -1.73 | 0.080 | 7 | 3 | 1.000 | 3.25 | 0.071 |
| WSB/EiJ | WSB | 53 | 31 | 838 | 1005 | -0.67 | 0.500 | **9** | 0 | **0.023** | 0.11 | 0.746 |

p-values in **bold** are smaller than 0.05; n of max lifespan in **bold** indicate the gender with the highest number of longest living individuals

**Table S2. Longevity parameters of female and male mice**

| **Sex** | **n** | **Days at death** | | | **Variation** | **Test difference sexes** | | | | | |
| --- | --- | --- | --- | --- | --- | --- | --- | --- | --- | --- | --- |
|  |  | **25%** | **50%** | **75%** | **MAD** | **Median** | | **Variation** | | **Log-rank** | |
|  |  |  |  |  |  | **z** | **p** | **z** | **p** | **χ²** | **p** |
| **Female** | 1,851 | 540.5 | 697 | 834 | 215 | -1.37 | 0.170 | -1.29 | 0.200 | 0.3 | 0.590 |
| **Male** | 950 | 532.3 | 709 | 840.8 | 226.8 |  |  |  |  |  |  |

**Table S3. Lifespan MAD of inbred strains**

| **Strain** | **Female** | **Male** | **Sex difference** |
| --- | --- | --- | --- |
| 129S1 | 199.4 | 144.6 | 54.9 |
| A | 170.5 | 129.7 | 40.8 |
| cBy | 131.2 | **264.6** | **-133.4** |
| BTBR | **87.5** | 196.4 | **-109.0** |
| BUB | **262.4** | **296.5** | -34.1 |
| C3H | 204.6 | 158.6 | 46.0 |
| B10 | **260.2** | 121.6 | **138.6** |
| B6 | 176.4 | 98.6 | 77.8 |
| BLKS | 167.5 | 94.1 | 73.4 |
| BRCD | **114.9** | 140.1 | -25.2 |
| C57L | **37.8** | **71.9** | -34.1 |
| CAST | **330.6** | **378.1** | -47.4 |
| CBA | **223.9** | 204.6 | 19.3 |
| D2 | **239.4** | 160.1 | 79.3 |
| FVB | **332.1** | 151.2 | **180.9** |
| KK | **68.2** | 115.6 | -47.4 |
| LP | 197.2 | 137.9 | 59.3 |
| MOLF | 163.8 | 157.2 | 6.7 |
| MRL | **112.7** | **68.2** | 44.5 |
| NOD.B10 | 135.7 | **269.8** | **-134.2** |
| NON | 123.1 | 97.1 | 25.9 |
| NZO | 202.4 | **259.5** | -57.1 |
| NZW | 196.4 | **292.1** | **-95.6** |
| P | 174.9 | 148.3 | 26.7 |
| PL | 150.5 | 144.6 | 5.9 |
| PWD | 199.4 | 176.4 | 23.0 |
| R3 | 127.5 | 119.3 | 8.2 |
| SJL | 174.9 | 146.8 | 28.2 |
| SM | 122.3 | **78.6** | 43.7 |
| SWR | **274.3** | **295.0** | -20.8 |
| WSB | **427.0** | **309.9** | **117.1** |

MAD in **grey** are very low, in **bold** very high; sex differences in **bold** are significant (p<0.05)

**Table S4. Association of maximum lifespan with circulating IGF1 levels at 6, 12, and 18 months**

| **Age (month)** | **Female** | | **Male** | | **Diff. sexes** | | **Model** | |
| --- | --- | --- | --- | --- | --- | --- | --- | --- |
|  | **Slope** | **p** | **Slope** | **p** | **Slope** | **p** | **adj. R²** | **p** |
| **6** | -0.35 | 0.066 | 0.65 | **0.018** | 1.00 | **0.002** | 0.11 | **0.020** |
| **12** | -0.33 | 0.136 | 0.67 | **0.042** | 1.00 | **0.010** | 0.07 | 0.071 |
| **18** | -0.07 | 0.780 | 1.25 | **<0.001** | 1.32 | **0.003** | 0.15 | **0.006** |

p-values in bold are smaller than 0.05

**Table S5A. Comparison of six-month IGF1 level between sexes**

| **Strain** | **IGF1 at 6 months** | | | | | | **Tests** | | | |
| --- | --- | --- | --- | --- | --- | --- | --- | --- | --- | --- |
|  | **Females** | | | **Males** | | | **Median** | | **Variance** | |
|  | **n** | **median** | **MAD** | **n** | **median** | **MAD** | **z** | **p** | **z** | **p** |
| LP/J* | 8 | 268.0 | 62.27 | 8 | 350.0 | 25.20 | -2.905 | **0.004** | 0.214 | 0.831 |
| CAST/EiJ* | 7 | 308.0 | 31.13 | 4 | 398.5 | 22.98 | -2.619 | **0.009** | 0.560 | 0.576 |
| 129S1/SvImJ | 8 | 264.5 | 47.44 | 8 | 300.0 | 28.91 | -1.937 | 0.053 | 0.287 | 0.774 |
| BALB/cByJ | 8 | 335.5 | 19.27 | 8 | 344.0 | 31.88 | -1.937 | 0.053 | -1.115 | 0.265 |
| CBA/J | 8 | 304.0 | 13.34 | 8 | 319.5 | 12.60 | -1.937 | 0.053 | 0.520 | 0.603 |
| DBA/2J | 8 | 247.0 | 35.58 | 8 | 287.5 | 28.17 | -1.937 | 0.053 | 0.324 | 0.746 |
| MRL/MpJ | 8 | 388.5 | 21.50 | 8 | 415.5 | 23.72 | -1.937 | 0.053 | -0.752 | 0.452 |
| SM/J | 8 | 171.5 | 63.01 | 8 | 231.0 | 15.57 | -1.937 | 0.053 | 1.663 | 0.867 |
| FVB/NJ | 8 | 267.0 | 44.48 | 8 | 308.5 | 15.57 | -1.464 | 0.143 | 1.360 | 0.174 |
| PL/J | 7 | 259.0 | 26.69 | 8 | 282.5 | 26.69 | -1.270 | 0.204 | 0.516 | 0.606 |
| KK/H1J | 8 | 402.5 | 54.86 | 4 | 434.0 | 17.79 | -1.173 | 0.241 | 0.501 | 0.616 |
| NZW/LacJ | 7 | 371.0 | 4.45 | 8 | 367.0 | 37.81 | -0.267 | 0.789 | -1.018 | 0.309 |
| WSB/EiJ | 7 | 228.0 | 34.10 | 6 | 231.0 | 15.57 | -0.247 | 0.805 | 1.741 | 0.082 |
| BUB/BnJ | 8 | 394.5 | 89.70 | 4 | 381.0 | 21.50 | 0.000 | 1.000 | 1.186 | 0.236 |
| MOLF/EiJ | 6 | 231.5 | 57.08 | 6 | 229.0 | 36.32 | 0.000 | 1.000 | 1.276 | 0.202 |
| PWD/PhJ | 8 | 344.5 | 68.94 | 8 | 330.0 | 14.08 | 0.000 | 1.000 | 1.098 | 0.272 |
| C57BL/6J | 8 | 254.5 | 35.58 | 7 | 258.0 | 13.34 | 0.267 | 0.789 | 1.532 | 0.126 |
| C57BL/10J | 16 | 260.0 | 21.50 | 8 | 253.0 | 21.50 | 0.848 | 0.397 | 0.044 | 0.965 |
| C3H/HeJ | 8 | 351.5 | 42.25 | 8 | 332.0 | 12.60 | 0.968 | 0.333 | 1.474 | 0.141 |
| C57BLKS/J | 8 | 294.5 | 22.24 | 8 | 276.5 | 15.57 | 0.968 | 0.333 | 1.024 | 0.306 |
| RIIIS/J | 8 | 290.0 | 43.00 | 8 | 269.0 | 17.79 | 0.968 | 0.333 | 2.023 | **0.043** |
| SWR/J | 8 | 380.5 | 43.74 | 8 | 358.5 | 60.79 | 0.968 | 0.333 | -1.035 | 0.301 |
| NON/ShiLtJ | 8 | 361.0 | 47.44 | 6 | 332.0 | 21.50 | 1.041 | 0.298 | 0.347 | 0.729 |
| A/J | 8 | 335.0 | 24.46 | 8 | 289.0 | 24.46 | 1.937 | 0.053 | 0.752 | 0.452 |
| P/J | 8 | 362.0 | 73.39 | 8 | 325.5 | 11.86 | 1.937 | 0.053 | 1.899 | 0.058 |
| C57BR/CDJ* | 5 | 372.0 | 25.20 | 4 | 334.5 | 26.69 | 2.263 | **0.024** | -1.112 | 0.266 |
| NZO/H1LtJ* | 8 | 444.5 | 41.51 | 7 | 386.0 | 32.62 | 2.272 | **0.023** | 0.577 | 0.564 |
| SJL/J* | 8 | 304.5 | 20.02 | 7 | 226.0 | 23.72 | 2.272 | **0.023** | 0.168 | 0.867 |
| BTBR<+>tf/J* | 8 | 447.0 | 28.91 | 8 | 367.5 | 39.29 | 2.905 | **0.004** | -0.368 | 0.713 |
| NOD.B10Sn* | 8 | 472.0 | 42.25 | 8 | 376.0 | 11.12 | 2.905 | **0.004** | 1.190 | 0.234 |
| C57L/J* | 8 | 374.5 | 35.58 | 7 | 319.0 | 14.83 | 3.274 | **0.001** | 1.597 | 0.110 |

p-values in **bold** are smaller than 0.05

**Table S5B. Comparison of 12-month IGF1 level between sexes**

| **Strain** | **IGF1 at 12 months** | | | | | | **Tests** | | | |
| --- | --- | --- | --- | --- | --- | --- | --- | --- | --- | --- |
|  | **Females** | | | **Males** | | | **Median** | | **Variance** | |
|  | **n** | **median** | **MAD** | **n** | **median** | **MAD** | **z** | **p** | **z** | **p** |
| C57BLKS/J* | 8 | 227.0 | 21.50 | 8 | 297.50 | 25.20 | -3.416 | **0.001** | -0.990 | 0.322 |
| C57BL/6J* | 8 | 227.5 | 17.79 | 8 | 265.50 | 21.50 | -2.905 | **0.004** | 0.296 | 0.767 |
| PL/J* | 7 | 231.0 | 17.79 | 5 | 256.00 | 8.90 | -2.180 | **0.029** | 1.106 | 0.269 |
| A/J* | 8 | 321.5 | 20.76 | 6 | 393.00 | 19.27 | -2.082 | **0.037** | 0.299 | 0.765 |
| CBA/J* | 8 | 308.0 | 44.48 | 6 | 365.50 | 11.86 | -2.082 | **0.037** | 0.449 | 0.653 |
| BUB/BnJ* | 3 | 297.0 | 28.17 | 5 | 357.00 | 25.20 | -2.049 | **0.040** | -0.450 | 0.653 |
| RIIIS/J | 8 | 251.5 | 74.87 | 8 | 286.50 | 36.32 | -1.937 | 0.053 | 1.768 | 0.077 |
| WSB/EiJ | 9 | 255.0 | 17.79 | 9 | 269.00 | 29.65 | -1.844 | 0.065 | 0.102 | 0.919 |
| FVB/NJ | 7 | 293.0 | 19.27 | 7 | 333.00 | 7.41 | -1.545 | 0.122 | 0.011 | 0.991 |
| MRL/MpJ | 7 | 323.0 | 75.61 | 8 | 349.50 | 26.69 | -1.270 | 0.204 | 1.403 | 0.161 |
| CAST/EiJ | 9 | 337.0 | 32.62 | 7 | 343.00 | 62.27 | -0.488 | 0.626 | -1.036 | 0.300 |
| 129S1/SvImJ | 7 | 312.0 | 40.03 | 8 | 313.50 | 31.88 | -0.267 | 0.789 | 0.598 | 0.550 |
| SJL/J | 7 | 263.0 | 16.31 | 8 | 269.00 | 24.46 | -0.267 | 0.789 | 0.348 | 0.728 |
| LP/J | 8 | 326.0 | 9.64 | 7 | 323.00 | 28.17 | -0.204 | 0.838 | -0.185 | 0.853 |
| C57BR/CDJ | 8 | 348.5 | 31.88 | 8 | 346.00 | 18.53 | 0.000 | 1.000 | 0.457 | 0.648 |
| NZO/H1LtJ | 6 | 417.0 | 7.41 | 4 | 373.50 | 80.80 | 0.000 | 1.000 | -2.586 | **0.010** |
| SM/J | 8 | 233.5 | 21.50 | 8 | 232.00 | 22.98 | 0.000 | 1.000 | 0.603 | 0.547 |
| NOD.B10Sn | 8 | 380.5 | 17.05 | 7 | 376.00 | 16.31 | 0.267 | 0.789 | -0.196 | 0.845 |
| KK/H1J | 7 | 319.0 | 94.89 | 7 | 266.00 | 56.34 | 0.515 | 0.607 | 1.041 | 0.298 |
| C57BL/10J | 7 | 257.0 | 32.62 | 8 | 235.00 | 17.79 | 0.735 | 0.462 | 1.967 | **0.049** |
| SWR/J | 7 | 380.0 | 45.96 | 8 | 342.50 | 19.27 | 0.735 | 0.462 | 0.604 | 0.547 |
| C3H/HeJ | 8 | 309.0 | 41.51 | 8 | 293.00 | 24.46 | 0.968 | 0.333 | 1.414 | 0.157 |
| BALB/cByJ | 8 | 356.5 | 32.62 | 7 | 345.00 | 32.62 | 1.270 | 0.204 | 0.540 | 0.590 |
| BTBR<+>tf/J | 8 | 470.0 | 54.11 | 7 | 416.00 | 80.06 | 1.270 | 0.204 | -1.068 | 0.286 |
| DBA/2J | 7 | 276.0 | 19.27 | 7 | 246.00 | 34.10 | 1.545 | 0.122 | 0.454 | 0.650 |
| NON/ShiLtJ | 7 | 399.0 | 22.24 | 8 | 366.50 | 45.22 | 1.737 | 0.082 | 0.020 | 0.984 |
| NZW/LacJ | 8 | 363.5 | 48.18 | 7 | 288.00 | 8.90 | 1.837 | 0.066 | 1.744 | 0.081 |
| PWD/PhJ* | 8 | 417.0 | 71.16 | 13 | 332.00 | 14.83 | 2.801 | **0.005** | 2.086 | **0.037** |
| C57L/J* | 8 | 401.5 | 36.32 | 8 | 299.50 | 22.98 | 2.905 | **0.004** | 1.041 | 0.298 |
| P/J* | 7 | 402.0 | 16.31 | 6 | 367.50 | 4.45 | 2.969 | **0.003** | 0.606 | 0.544 |

p-values in **bold** are smaller than 0.05

**Table S5C. Comparison of 18-month IGF1 level between sexes**

| **Strain** | **IGF1 at 18 months** | | | | | | **Tests** | | | |
| --- | --- | --- | --- | --- | --- | --- | --- | --- | --- | --- |
|  | **Females** | | | **Males** | | | **Median** | | **Variance** | |
|  | **n** | **median** | **MAD** | **n** | **median** | **MAD** | **z** | **p** | **z** | **p** |
| MRL/MpJ* | 8 | 203.5 | 19.3 | 8 | 269.5 | 47.4 | -2.905 | **0.004** | -1.108 | 0.268 |
| WSB/EiJ* | 8 | 240.0 | 10.4 | 9 | 283.0 | 44.5 | -2.611 | 0.009 | -0.499 | 0.618 |
| C3H/HeJ* | 8 | 280.0 | 16.3 | 8 | 325.5 | 23.7 | -2.440 | **0.015** | 0.387 | 0.699 |
| RIIIS/J* | 8 | 208.5 | 71.9 | 8 | 293.0 | 25.9 | -2.440 | **0.015** | 2.374 | **0.018** |
| LP/J* | 7 | 309.0 | 14.8 | 8 | 351.0 | 16.3 | -2.272 | **0.023** | 0.338 | 0.736 |
| BUB/BnJ* | 11 | 306.0 | 66.7 | 6 | 362.0 | 27.4 | -2.147 | **0.032** | 1.750 | 0.080 |
| A/J | 7 | 303.0 | 23.7 | 7 | 329.0 | 26.7 | -1.545 | 0.122 | -0.653 | 0.514 |
| DBA/2J | 5 | 215.0 | 20.8 | 6 | 258.5 | 31.1 | -1.476 | 0.140 | -0.313 | 0.754 |
| FVB/NJ | 7 | 267.0 | 50.4 | 4 | 338.0 | 31.9 | -1.418 | 0.156 | 0.117 | 0.907 |
| C57BLKS/J | 7 | 252.0 | 31.1 | 8 | 279.5 | 14.8 | -1.270 | 0.204 | 1.186 | 0.236 |
| C57BL/10J | 7 | 251.0 | 46.0 | 7 | 274.0 | 25.2 | -0.515 | 0.607 | 1.154 | 0.248 |
| NZW/LacJ | 7 | 379.0 | 44.5 | 6 | 380.5 | 14.8 | -0.247 | 0.805 | 1.770 | 0.077 |
| NON/ShiLtJ | 6 | 354.0 | 62.3 | 7 | 351.0 | 54.9 | 0.247 | 0.805 | 0.442 | 0.659 |
| 129S1/SvImJ | 7 | 310.0 | 40.0 | 14 | 317.0 | 17.0 | 0.310 | 0.757 | 0.932 | 0.352 |
| KK/H1J | 7 | 303.0 | 31.1 | 7 | 265.0 | 56.3 | 0.515 | 0.607 | 0.068 | 0.946 |
| SWR/J | 7 | 332.0 | 11.9 | 7 | 325.0 | 32.6 | 0.515 | 0.607 | -0.647 | 0.577 |
| C57BL/6J | 7 | 298.0 | 32.6 | 8 | 275.0 | 7.4 | 0.735 | 0.462 | 1.990 | **0.047** |
| CBA/J | 9 | 315.0 | 44.5 | 6 | 304.0 | 20.0 | 0.817 | 0.414 | 1.799 | 0.072 |
| BALB/cByJ | 8 | 369.0 | 27.4 | 8 | 287.5 | 64.5 | 0.968 | 0.333 | -1.353 | 0.176 |
| C57BR/CDJ | 8 | 325.5 | 40.0 | 8 | 301.0 | 35.6 | 0.968 | 0.333 | 0.511 | 0.610 |
| SM/J | 6 | 251.5 | 28.2 | 9 | 224.0 | 34.1 | 1.225 | 0.221 | 0.558 | 0.577 |
| SJL/J | 6 | 283.5 | 34.1 | 5 | 243.0 | 11.9 | 1.476 | 0.140 | 1.300 | 0.194 |
| PWD/PhJ | 7 | 341.0 | 56.3 | 7 | 280.0 | 20.8 | 1.545 | 0.122 | -0.228 | 0.819 |
| BTBR<+>tf/J | 7 | 354.0 | 17.8 | 6 | 314.0 | 39.3 | 1.897 | 0.058 | -0.990 | 0.322 |
| P/J* | 7 | 368.0 | 41.5 | 4 | 315.5 | 11.1 | 2.182 | **0.029** | 0.699 | 0.485 |
| NOD.B10Sn* | 6 | 437.5 | 24.5 | 9 | 383.0 | 50.4 | 2.245 | **0.025** | -0.676 | 0.499 |
| PL/J* | 6 | 255.5 | 21.5 | 7 | 231.0 | 31.1 | 2.392 | **0.017** | -0.467 | 0.641 |
| NZO/H1LtJ* | 7 | 433.0 | 44.5 | 7 | 344.0 | 34.1 | 2.575 | **0.010** | 0.173 | 0.862 |
| CAST/EiJ* | 7 | 363.0 | 11.9 | 8 | 319.0 | 33.4 | 2.739 | **0.006** | -1.817 | 0.069 |
| C57L/J* | 7 | 321.0 | 32.6 | 6 | 287.5 | 10.4 | 2.969 | **0.003** | 1.577 | 0.115 |

p-values in **bold** are smaller than 0.05
