# Supplementary material for: Genetic differences and longevity‐related phenotypes influence lifespan and lifespan variation in a sex‐specific manner in mice
Source: Aging Cell. 2020 Oct 26;19(11):e13263. doi: 10.1111/acel.13263 (PMC7681063; doi:10.1111/acel.13263)
Supplement: Supplementary file 9 [file ACEL-19-e13263-s009.docx]

**Figure S1. Comparison of survival curves and lifespan between female and male mice.** Comparing the survival curves of all female and male mice (1,851 and 950, respectively), ignoring the strain difference, the log rank test shows no significant difference between female and male mice (A). t test of the lifespan comparison between sexes shows there is no significant difference.

**Figure S2. Comparison of survival curves** between strains with high and low IGF1 levels (A & B), and between the domesticated and wild-derived inbred strains (C & D), females and male are shown separately. Log rank tests show all the comparisons are significantly different (p < 0.05). The strain names embedded in the A and B are for the strains with high or low IGF1 levels that were identified previously (Yuan R, 2009).

**Figure S3. Association of maximum lifespan with circulating IGF1 levels** at ages of 6 (A), 12 (B) and 18 (C) months. In females, at all three time points, the associations are negative but none of them is significant. In males, at all three time points, the associations are positive and significant (p < 0.05). The differences between sexes are significant at all three time points (p < 0.05). The slopes and p values are listed in Table S4.

**Figure S4. Comparison of median lifespan between domesticated and wild-derived inbred strains,** female (A) and male (B) separately**.** No significant difference is detected**.** p-values are of the median-test.
